# Zinc promotes cell apoptosis via activating the Wnt-3a/β-catenin signaling pathway in osteosarcoma

**DOI:** 10.1186/s13018-020-01585-x

**Published:** 2020-02-19

**Authors:** Kai Gao, Yingchun Zhang, Jianbing Niu, Zhikui Nie, Qingsheng Liu, Chaoliang Lv

**Affiliations:** 1 Department of Orthopedics, Jining No.1 People’s Hospital, Jining, China; 2 Department of Interventional Radiology, Jining No.1 People’s Hospital, Jining, China

**Keywords:** Osteosarcoma, Zinc, Wnt/β-catenin, Apoptosis

## Abstract

**Background:**

The zinc content in the blood and tumor tissues of patients with osteosarcoma and the underlying regulation and molecular mechanism of zinc have not been reported.

**Methods and results:**

This study showed that the zinc content in the blood and tumor tissues of patients with osteosarcoma significantly reduced. CCK-8 and Transwell chamber assays revealed that zinc treatment significantly inhibited the proliferation and invasion abilities of osteosarcoma cells. Western blot analysis indicated that the expression levels of caspase-3 and caspase-9 were significantly increased, suggesting that zinc inhibited the growth and promoted the apoptosis of osteosarcoma cells. In addition, the expression levels of Wnt-3a and β-catenin, the marker proteins of the Wnt/β-catenin signaling pathways, were significantly increased in osteosarcoma cells after zinc intervention, which demonstrated that the pathway was clearly activated. However, the effect of zinc on the apoptosis, proliferation, and invasion abilities of osteosarcoma cells was reversed when the Wnt/β-catenin signaling pathways was inhibited by XAV939 (Wnt antagonist) treatment.

**Conclusions:**

This study is the first to report the changes in zinc levels in the blood and tumor tissues of patients with osteosarcoma and to preliminarily verify that zinc inhibits the proliferation and invasion and promote the apoptosis of osteosarcoma cells by inducing the Wnt/β-catenin signaling pathway, which ultimately inhibit cancer growth.

## Introduction

Osteosarcoma (OS) is a malignant tumor that features insidious onset, rapid growth, excessive proliferation of tumor cells, and distant invasion and metastasis [1, 2]. At present, the treatment of osteosarcoma mainly includes immunotherapy (adoptive immunotherapy, active specific immunotherapy, and non-specific immunotherapy), gene therapy, targeted therapy, and chemotherapy [3, 4]. However, the specific mechanism of osteosarcoma remains unclear, and effective prevention and treatment measures for this disease have not been found.

Zinc is an essential trace element in the human body [5]. It is closely related to many physiological activities, such as cell growth and differentiation and gene transcription [6, 7]. Zinc deficiency seriously affects the normal physiological functions of the body. Some studies found that zinc in trace elements is associated with the occurrence and development of malignant tumors [8]. In addition, a significant negative correlation exists between the intake of zinc in the diet and the incidence of cancer in the digestive tract, especially colorectal cancer [9]. The zinc content in the serum of patients with gastric cancer is significantly reduced [10], and zinc in breast cancer plays an important role in cell proliferation [11]. However, the zinc content in the blood and tumor tissues of patients with osteosarcoma and the underlying regulation and molecular mechanism of zinc have not been elucidated.

In our study, we will firstly observe the difference of zinc in clinical patients with osteosarcoma, and then further verify the regulation of zinc on osteosarcoma and the molecular mechanism in the level of osteosarcoma cell culture. The successful development of this study will provide a novel regulatory factor and molecular mechanism for the study of osteosarcoma, promote the study of osteosarcoma, and promote the clinical transformation of zinc on osteosarcoma.

## Materials and methods

### General information

Forty-five patients with osteosarcoma and 45 healthy volunteers were enrolled in Jining No.1 People’s Hospital from July 2013 to July 2018. All patients were diagnosed with osteosarcoma via surgical pathological biopsy (CT-guided multi-target, minimally invasive puncture biopsy). The general clinical data were as follows: 1) patients with osteosarcoma, 32 males and 13 females; aged 10.5–22.3 years, mean age (17.37 ± 5.21 years old); body weight 20.12–53.15 kg, average body weight (40.55 ± 12.23 kg); osteosarcoma tissue type, 16 cases of tibia, 15 cases of femur, 14 cases of humerus; and Enneking clinical stage, 8 cases of stage I, 15 cases of stage II A, 17 cases of stage II B, and 5 cases of stage III; 2) healthy volunteers, 30 males and 15 females; aged 10.0–24.5 years, mean age (18.18 ± 4.33 years old); body weight 19.33–55.32 kg, average body weight (41.56 ± 13.29 kg). General information for osteosarcoma patients and healthy volunteers was not statistically significant, *p* > 0.05. All subjects were informed of the research content and voluntarily signed informed consent. All experiments were in accordance with The Code of Ethics of the World Medical Association (Declaration of Helsinki).

### Atomic absorption spectrometry

The zinc content in the blood of normal people and osteosarcoma patients was detected by atomic absorption spectrometry. Fasting venous blood (1 mL) was extracted from normal people and osteosarcoma patients in the morning. Then, a 40 μL aliquot of the blood sample was added to the human element assay kit liquid (Turatong, strontium chloride, bovine serum, and pure water) and mixed thoroughly. After standing at room temperature for 30 min, the suspension was placed in an atomic absorption spectrometer for detection.

### Inductively coupled plasma mass spectrometry

Exactly 10 g each of tumor central tissue, adjacent tissues, and normal tissues was obtained from osteosarcoma patients. After homogenization of the tissue, 1 g of each group of samples was placed in a microwave digestion inner tank, added with 10 mL of nitric acid, and stored in the dark at room temperature. Overnight, standard tissue digestion was performed in accordance with the following protocol of the microwave digestion instrument: 120 °C, heating time for 5 min and then constant temperature for 5 min; 150 °C, heating time for 5 min and then constant temperature for 10 min; 190 °C, heating time for 5 min and then constant temperature for 20 min. Then, the tissue was cooled, the can lid was slowly opened, and the digestion tank was placed in an ultrasonic water bath and ultrasonically degassed for 5 min. The volume was adjusted to 50 mL with water, and the mixed liquid was placed in an inductively coupled plasma mass spectrometer to detect the zinc content in the tissues of the three groups.

### Cell culture

Human osteosarcoma cell line U-2OS was purchased from Shanghai Cell Bank of Chinese Academy of Sciences (Shanghai, China). The cells were cultured in DMEM medium containing 10% fetal bovine serum (FBS; Vian-saga, Shanghai, China), 100 μg/mL penicillin, and 100 μg/mL streptomycin. The cells were cultured in a constant temperature and humidity incubator at 5% CO_2_ and 37 °C for logarithmic growth. The cells were divided into three groups: Control, Saline, and ZnSO_4_ (Sigma-Aldrich) group and then four groups: Control, Saline, ZnSO_4_ and ZnSO_4_ + XAV939 (Wnt antagonist [12]) group. After 24 h of treatment, the cells were used for Western blot, CCK-8 assay, and Transwell chamber methods.

### MTT assay

The cell viability for subsequent ZnSO_4_ (0, 10, 20, 30, 40, 50, 60, 70, 80, 90, 100, and 200 μM) treatment was detected by the reduction in 3-(4,5-dimethylthiazol-2-yl)-2,5-diphenyltetrazolium bromide to a purple formazan product (MTT) assay. In brief, MTT (20 μL, Sigma-Aldrich) was added to each well, and the U-2OS cells were incubated at 37 °C for 4 h. The media were decanted, and 150 μL of dimethyl sulfoxide (Sigma-Aldrich) was added to each well. Finally, the absorbency at 490 nm was determined on a Thermo Scientific™ Varioskan™ Flash Multimode Reader (Thermo Fisher Scientific, Inc., Waltham, MA, USA).

### Western blot

After 24 h of U-2OS cell culture, BCA protein concentration was measured to, and the final configuration of each sample concentration was 2 μg/μL. The protein was transferred onto a PVDF membrane after electrophoresis. Then, the protein was sealed for 2 h at room temperature with a closed solution. Anti-Wnt-3a (1:1000; Cell Signaling Technology, Inc.), anti-β-catenin (1:500; Cell Signaling Technology, Inc.), anti-cleaved-caspase-9 (1:1000; Novus Biologicals), anti-cleaved-caspase-3 (1:1000; Novus Biologicals), and anti-β-actin (1:2000, Abcam, Cambridge, UK) were incubated overnight at 4 °C diluted by a hybridization solution. The next day, the PVDF films were hybridized at room temperature with goat anti-rabbit IgG (1:2000, Abcam, Cambridge, UK) diluted by hybridization solution for 2 h and then imaged on the UVP gel electrophoresis system (UVP, LLC, Upland, CA, USA). The gray value of the target protein was compared with the gray value of β-actin.

### CCK-8 assay

The U-2OS cells were divided into different groups. After incubation for 24 h, the cells were added with 10 μL of CCK-8 reagent (Sigma-Aldrich) and incubated at 37 °C for 2 h. The optical density (OD) value of the cells was measured at 450 nm using a microplate reader. The larger the OD value, the stronger the cell proliferation ability.

### Transwell chamber method

U-2OS cells were cultured for 24 h and then inoculated into a 24-well Transwell chamber pre-coated with basement matrigel (Sigma-Aldrich), and the upper chamber inoculation with 200 μL of cell suspension prepared in serum-free medium. The medium containing 10% fetal bovine serum was added to the lower chamber and then incubated for 48 h at 37 °C. The chamber was removed, and the matrigel and cells in the upper chamber were wiped off with a cotton swab. The cells were fixed with 4% paraformaldehyde for 30 min, washed three times with phosphate buffer solution, stained with 0.1% crystal violet for 20 min, and then washed three times with water. The number of cell-penetrating cells (purple red cells) was observed under a microscope (Leica CM3050S; Heidelberg, Germany) at × 100 magnification. Five fields were randomly selected for viewing count, and the average. The greater the number of transmembrane cells, the stronger the invasive ability.

### Statistical analysis

All data were expressed as mean ± SD and analyzed using the Graph Prism Program, Version 5.0 (GraphPad Software, Inc., La Jolla, USA). Unpaired Student’s *t* test and one-way ANOVA separately was used to test the comparison among groups and the multiple groups. Statistical significance was considered at *p* < 0.05.

## Results

### Blood zinc content of patients with osteosarcoma is reduced

**Fig. 1:**
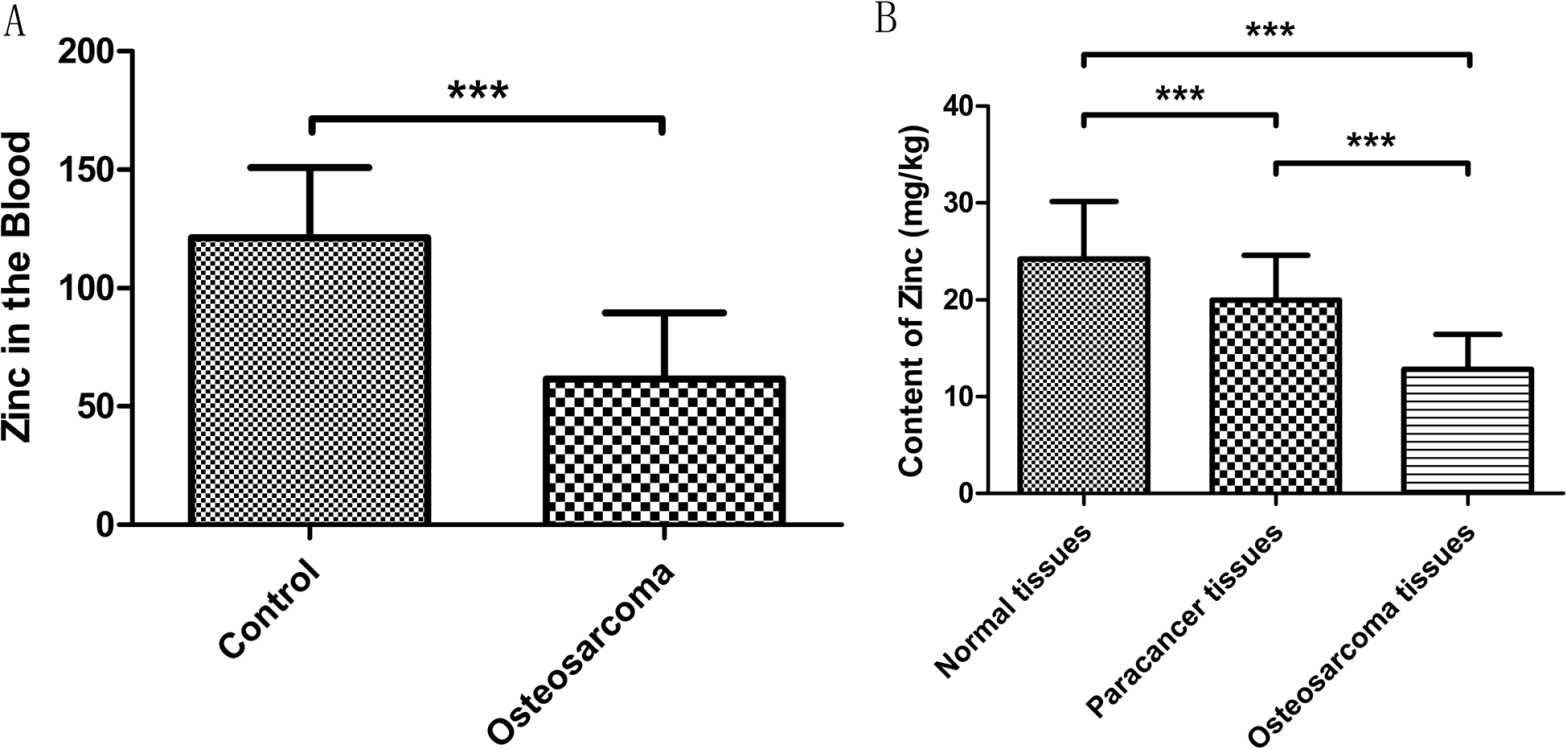
Blood zinc content (**a**) and tissue zinc (**b**) of patients with osteosarcoma was quantitatively detected via atomic absorption spectrometry and ICP-MS. ***p < 0.001 in comparison between groups. *n* = 45 per group

In clinical trials, blood was collected from normal and osteosarcoma patients, and changes in zinc content were determined by atomic absorption spectrometry (Fig. 1a). The zinc ion content in the blood of patients with osteosarcoma was significantly lower than that in the blood of normal people. This result indicates that patients with osteosarcoma have zinc deficiency.

### Zinc content in osteosarcoma tissues is significantly reduced

Normal, paracancerous, and cancer tissues were selected from patients with osteosarcoma, and the zinc content in the three tissues was detected by ICP-MS (Fig. 1b). Compared with that in normal tissues, the zinc content in paracancerous tissues was lower. The zinc content in cancer tissues was also significantly lower than those in normal and adjacent tissues. The above results confirmed that the zinc content in osteosarcoma patients is significantly reduced in blood and tissues, indicating that zinc deficiency could be a “potential” factor in the pathogenesis of the disease.

### Selection of concentrations for ZnSO_4_ treatment in U-2OS cells

**Fig. 2:**
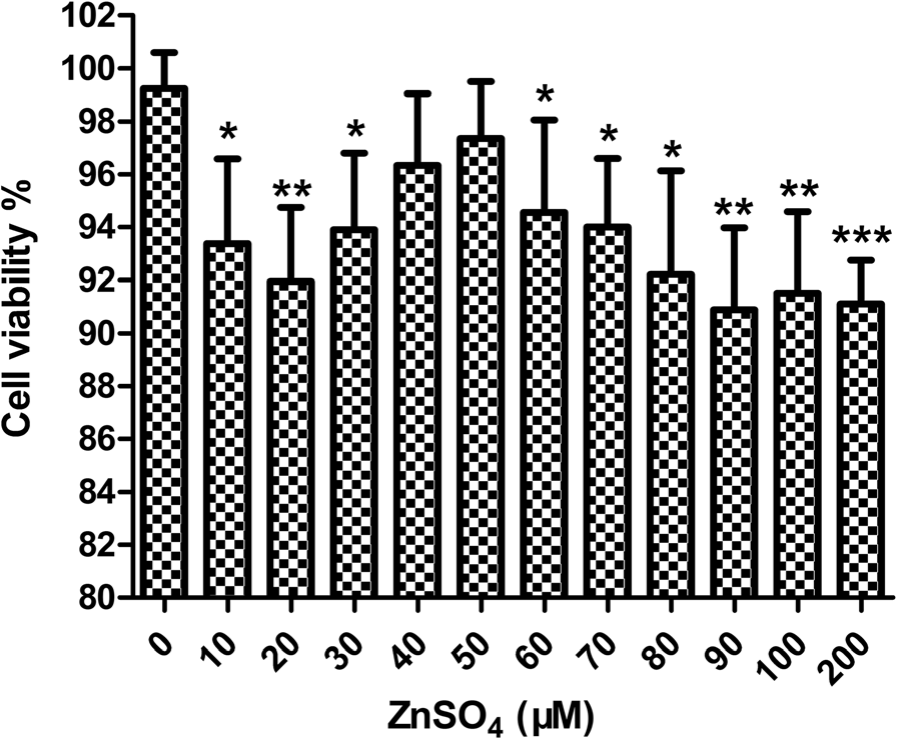
Selection of concentrations for ZnSO_4_ treatment in U-2OS cells via MTT assay. *p < 0.05, **p < 0.01, ***p < 0.001 compared with the control group. *n* = 4 per group

U-2OS cells were incubated with a series of ZnSO_4_ concentrations (0, 10, 20, 30, 40, 50, 60, 70, 80, 90, 100, and 200 μM) for 24 h, and the MTT assay was utilized to detect cell viability (Fig. 2). Treatment with ZnSO_4_ at 40 and 50 μM exerted no detrimental effects on the cells. Therefore, ZnSO_4_ at 40 μM was used in all subsequent experiments.

### Zinc inhibits the proliferation and invasion of osteosarcoma cells and promotes cell apoptosis by activating the Wnt/β-catenin signaling pathway

**Fig. 3:**
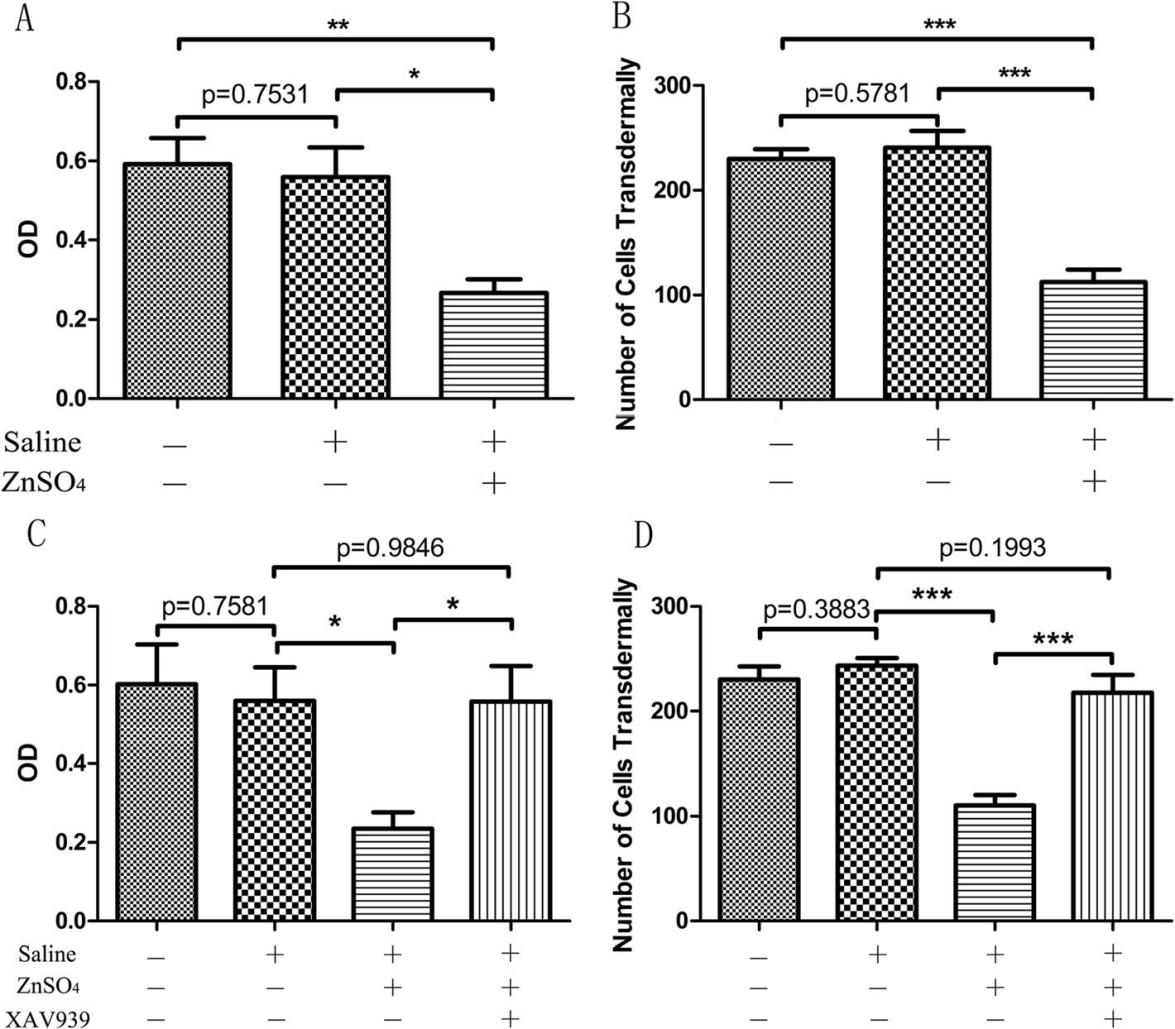
Proliferation and invasion of osteosarcoma cells were detected by CCK-8 (**a** and **c**) and Transwell chamber assays (**b** and **d**) in U-2OS cells. *p < 0.05, **p < 0.01, ***p < 0.001 in comparison between groups. *n* = 4 per group

CCK-8 (Fig. 3a) and Transwell chamber assays (Fig. 3b) were used to detect the regulatory effect of zinc on the proliferation and invasion abilities of cells. Results showed that the optical density (OD) of the osteosarcoma cells significantly decreased after ZnSO_4_ intervention. The number of transmembrane cells in the ZnSO_4_ group also significantly reduced compared with those in the control and saline groups. This result indicates that zinc can significantly inhibit the proliferation and invasive abilities of osteosarcoma cells.

**Fig. 4:**
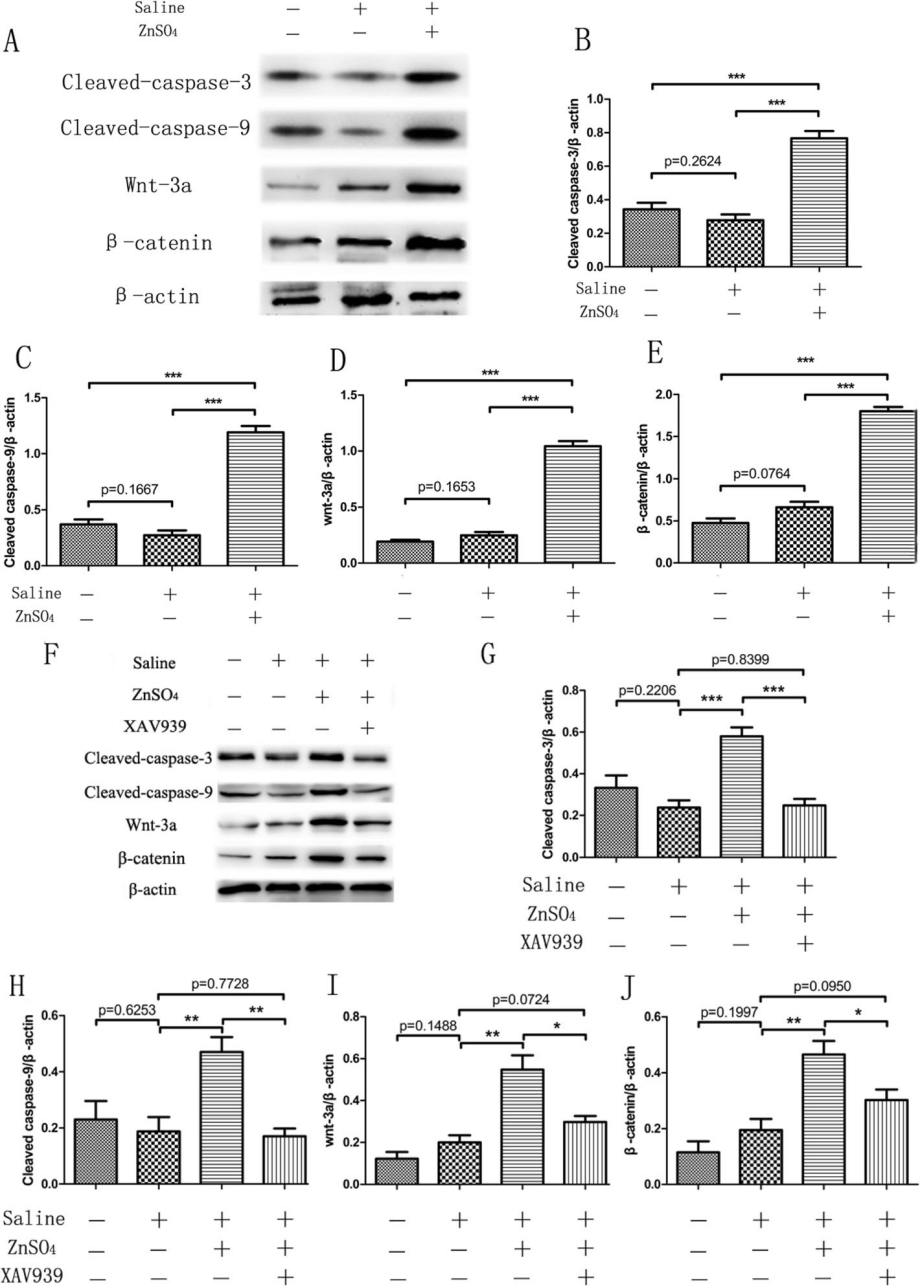
Expression levels of proteins of the apoptotic (**b**, **c**, **g** and **h**) and Wnt/β-catenin signaling pathways (**d**, **e**, **i** and **j**) were detected via western blot (**a** and **f**). *p < 0.05, **p < 0.01, ***p < 0.001 in comparison between groups. *n* = 4 per group

To further observe the regulation of zinc on osteosarcoma cells, we cultured U-2OS cells for 24 h and then used western blot to detect the apoptosis of cells (Fig. 4a). The expression levels of the apoptotic proteins caspase-3 (Fig. 4b) and caspase-9 (Fig. 4c) showed no significant difference between the control and saline groups. Compared with the above two groups, the expression levels of caspase-3 and caspase-9 significantly increased in the ZnSO_4_-treated cells, indicating that zinc promoted the apoptosis of osteosarcoma cells. Besides, the expression levels of Wnt-3a (Fig. 4d) and β-catenin (Fig. 4e) proteins, the marker protein of Wnt/β-catenin signaling pathway, were also obviously promoted, which implied the Wnt/β-catenin signaling pathway was activated by zinc in osteosarcoma cells.

Furthermore, the CCK-8 (Fig. 3c), Transwell chamber assays (Fig. 3d), and western blot (Fig. 4f) were further used to verify the molecular mechanism by which zinc affects osteosarcoma cells in the four groups (Control, Saline, ZnSO4, and ZnSO4 + XAV939 group). The results showed that zinc inhibited the proliferation and invasion of osteosarcoma cells, promoted cell apoptosis (Fig. 4g, h) and activated the Wnt/β-catenin signaling pathway (Fig. 4i, j); however, the effect of zinc on the apoptosis, proliferation, and invasion abilities of osteosarcoma cells was reversed when the Wnt/β-catenin signaling pathways was inhibited by XAV939 (Wnt antagonist) treatment (Fig. 4g, h, i, j). This result preliminarily verified that zinc exerts its anticancer effect by activating the Wnt/β-catenin signaling pathway.

## Discussion

In our study, we first found that the zinc content in the blood and tissues of patients with osteosarcoma is significantly reduced. Besides, we determined the effect of zinc treatment on osteosarcoma cells. Results showed that zinc treatment exerts its anticancer effect by significantly inhibiting the proliferation and invasive abilities and promoting the apoptosis of osteosarcoma cells through activating the Wnt/β-catenin signaling pathway. This study suggests zinc deficiency may be a potential factor, and zinc supplementation may be a novel preventive and therapeutic approach for osteosarcoma disease.

Osteosarcoma is a primary malignant tumor common in adolescents or children under the age of 20. This disease has the following clinical features: strong invasiveness, poor prognosis, and high metastatic rate [13–17]. Although osteosarcoma is treated in a variety of ways, local tumor recurrence, metastasis, and multi-drug resistance after surgery and low rate of tumor necrosis at the time of the surgery seriously hamper the postoperative condition and prognosis of osteosarcoma patients [18, 19]. Therefore, exploring new and effective, preventive and therapeutic measures, and in-depth studying of the molecular mechanism underlying osteosarcoma development are important to improve the status of osteosarcoma treatment.

Trace elements have special physiological functions in carrying and regulating body fluid osmotic pressure and acid–base balance, which are important components and active substances of enzymes [20]. Zinc is an important trace element in the human body, which promotes the growth and tissue regeneration of the body, participates in nucleic acid synthesis, inhibits lipid peroxidation, stabilizes cell membrane function, and alleviates damage by free radical peroxidase. Decreased zinc content can reduce the body’s immune mechanism, weaken the resistance of cells to free radicals and peroxides produced by cancer, and allow cells to divide and multiply in an unrestricted manner, causing cancer. Insufficient zinc can seriously affect the body’s normal physiological function [7, 21–23].

Zinc in human trace elements correlates with the occurrence and development of malignant tumors, such as rectal cancer, gastric cancer, and breast cancer. Zinc imbalance also affects the occurrence of head and neck cancer [24]. Zinc oxide nanoparticles exert selective cytotoxicity against tumor cells and can serve as an innovative antitumor agent [25]. Zinc can induce the apoptosis of PC-3 prostate cancer cells [26]. Zinc oxide nanoparticles provide potential anticancer activity toward tongue cancer cells [27]. However, changes in the levels of the trace element zinc in the blood and tissues of patients with osteosarcoma and the regulatory effect of zinc on osteosarcoma cells have not been reported. In our clinical work, the zinc content in the blood and tumor tissues of osteosarcoma patients was quantitatively detected. Results showed that the zinc content in the blood of patients with osteosarcoma was significantly lower than that in normal people. Zinc in osteosarcoma tissues was also significantly reduced, which initially verified the lack of zinc in osteosarcoma patients.

To further observe the regulatory effect of zinc on osteosarcoma, we cultured osteosarcoma cells in vitro. Treatment with zinc exerted its anticancer effect by significantly inhibiting the proliferation and invasion abilities and promoting the apoptosis of osteosarcoma cells. The above results further verified that zinc supplementation may prevent osteosarcoma and be potentially effective for treating osteosarcoma. However, the specific molecular mechanisms underlying the anticancer effects of zinc on osteosarcoma have not yet been elucidated.

Recent studies have found that the Wnt/β-catenin signaling pathway plays an important role in the occurrence and development of tumors, such as in the malignant transformation of the intestinal epithelium [28], hyperplasia of breast tissue [29], and in skin cancer [30]. In addition, HNF1A-AS1 exerts its role in the treatment of osteosarcoma by inducing the Wnt/β-catenin signaling pathway [31]. Plumbagin increases the value of osteosarcoma cells by interfering with the Wnt/β-catenin signaling pathway [32]. Meanwhile, microRNA-152 inhibits the growth of osteosarcoma cells by activating the Wnt/β-catenin signaling pathway [33], whereas histone methyltransferase SETD2 inhibits the growth of osteosarcoma cells by inhibiting the Wnt/β-catenin signaling pathway [34]. However, the specific role of the Wnt/β-catenin signaling pathway in osteosarcoma has not been validated.

In our experiments, we observed the specific molecular mechanism by which zinc regulates osteosarcoma. We cultured osteosarcoma cells in vitro and treated them with ZnSO_4_. Results showed that zinc treatment significantly inhibited the proliferation and invasion of osteosarcoma cells, promoted cell apoptosis, increased the expression levels of Wnt-3a and β-catenin, marker proteins of the Wnt/β-catenin signaling pathway, in osteosarcoma cells. The results preliminarily verified that zinc activated the Wnt-3a/β-catenin signaling pathway of osteosarcoma cells and played an anti-cancer effect. Meaningfully, we found that the effect of zinc on the apoptosis, proliferation, and invasion abilities of osteosarcoma cells was reversed when the Wnt/β-catenin signaling pathways were inhibited by XAV939, which verified that zinc exerts its anticancer effect by activating the Wnt/β-catenin signaling pathway. However, the anticancer effect and mechanism of zinc were preliminarily verified only by the change in proliferation and invasive abilities and by the expression of apoptotic proteins and Wnt-3a/β-catenin signaling pathway proteins in osteosarcoma cells. In our next experiments, we will verify the anticancer effect of zinc by combining cell, animal, and human experiments and elaborate the specific molecular mechanism by which zinc exerts its antitumor effect through the Wnt-3a/β-catenin signaling pathway.

## Conclusion

Zinc in the blood and tumor tissues of osteosarcoma patients is significantly deficient. Zinc exerted its anticancer effect by inhibiting the proliferation and invasion and promoting the apoptosis of osteosarcoma cells through activating the Wnt-3a/β-catenin signaling pathway. This innovative discovery may provide a potential preventive and therapeutic target for osteosarcoma and promote basic and clinical research of osteosarcoma.

## Data Availability

All data generated or analyzed during this study are included in the manuscript.

## Abbreviations

FBS: Fetal bovine serum
ICP-MS: Inductively coupled plasma mass spectrometry
MTT: 3-(4,5-dimethylthiazol-2-yl)-2,5-diphenyltetrazolium bromide to a purple formazan product
OD: Optical density
OS: Osteosarcoma
